# Comparative Analysis of Substrate-Free Cultured Oral Mucosal Epithelial Cell Sheets from Cells of Subjects with and without Stevens—Johnson Syndrome for Use in Ocular Surface Reconstruction

**DOI:** 10.1371/journal.pone.0147548

**Published:** 2016-01-25

**Authors:** Yun Hee Kim, Dong Hyun Kim, Eun Jung Shin, Hyun Ju Lee, Won Ryang Wee, Saewha Jeon, Mee Kum Kim

**Affiliations:** 1 Cutigen Research Institute, Tego Science Inc., Seoul, Korea; 2 Department of Ophthalmology, Seoul National University College of Medicine, Seoul, Korea; 3 Laboratory of Corneal Regenerative Medicine and Ocular Immunology, Seoul Artificial Eye Center, Seoul National University Hospital Biomedical Research Institute, Seoul, Korea; University of Newcastle upon Tyne, UNITED KINGDOM

## Abstract

**Purpose:**

To compare the regenerative potential of cultured oral mucosal epithelial cells sheets (COMECs) from Stevens-Johnson syndrome (SJS) subjects with those from non-SJS subjects.

**Methods:**

Human oral mucosal epithelial cells from SJS and non-SJS subjects were cultured, and colony-forming efficiency (CFE), proliferative and migration potential, expression of cytokines/growth factors and stem cells were compared. COMECs from SJS and non-SJS subjects were transplanted into 12 limbal stem cell-deficient rabbits, and their regenerative potential was analyzed at 1 week after transplantation.

**Results:**

CFE (p>0.05, student’s t test), cell proliferation potential (p>0.05, two-way ANOVA) and expression of the cytokeratins (K3, K4, K13, K19) in the oral mucosal epithelial cells from SJS subjects were similar to those of the cells from non-SJS subjects. The initial migratory potential of SJS cells was delayed compared to that of non-SJS cells (p <0.05, RM two-way ANOVA). The SJS cells expressed lower levels of EGF and higher levels of VEGF compared to that of non-SJS cells (p<0.05, one-way ANOVA). *In vivo* transplanted SJS-COMECs showed similar expression of K3, K4, and K13, proliferation markers (Ki-67; p>0.05, Mann-Whitney U test), and stem cell markers (p63; p>0.05, Mann-Whitney U test) compared to non-SJS COMECs. The initial epithelial defects *in vivo* were larger in the eyes treated with SJS-COMECs on day 3 (p<0.01, RM two-way ANOVA), but no differences were observed by day 7 between SJS- and non-SJS-COMECs.

**Conclusions:**

These results suggest that, aside from differences in migratory potential, oral mucosal epithelial cells from SJS and non-SJS subjects are comparable in their regeneration potential in treating limbal stem cell deficiency.

## Introduction

Total limbal stem cell deficiency is an intractable chronic ocular surface disease that causes blindness. Since Pellegrini et al. introduced autologous cultured limbal epithelial sheet transplantation for the treatment of chemically injured eyes [1], cell sheets from various cell sources and carriers have been used to treat limbal stem cell deficiency [2–6]. Currently, oral mucosal epithelial cells can be used to treat damaged ocular surfaces because they are readily available and have a phenotype similar to that of corneal epithelial cells [7, 8].

Stevens—Johnson syndrome (SJS) is a common cause of bilateral total limbal stem cell deficiency [9, 10]. Depending on the severity of the condition, the mucosal epithelium of the eye, oral cavity, GI tract, and genital tract may be affected. After systemic inflammation subsides, most epithelial tissues return to normal, with the exception of ocular tissue. Inflammation destroys the limbal stem cells of the eyes [11]. It is not known whether characteristics such as stemness of the oral mucosal epithelial cells from SJS subjects are similar to those of healthy subjects when inflammation is present in the oro-mucosal area.

Some ophthalmologists believe that oral mucosal epithelial sheets from SJS subjects may be more fragile than sheets from limbal stem cell-deficient patients who have normal oral cavities. Sotozono et al. reported frequent, persistent epithelial defects in the eyes of SJS patients transplanted with oral mucosal epithelial cells [12]. It is likely that characteristics of epithelial cells or stemness may be affected by severe inflammation in SJS. In fact, levels of Toll-like receptor 5 increased in conjunctival epithelial cells of SJS subjects compared with those in healthy subjects [13], suggesting that some cellular properties may be altered.

Hence, we investigated whether characteristics of the oral mucosal epithelial cells of SJS subjects such as stemness, proliferation and migration potential, and expression of cytokeratin and cytokines might differ from those of normal subjects.

## Materials and Methods

This study was performed in accordance with the guidelines of the Declaration of Helsinki. The clinical protocols were approved by the institutional review board of Seoul National University Hospital (IRB number: H-0707-043-213), and written informed consent was obtained from all participants. Informed consent documents were kept on file. All procedures used in this animal study were adhered to the ARVO Statement regarding the Use of Animals in Ophthalmic and Vision Research. The animal study protocol was approved by the Research Ethics Committee at Seoul National University Hospital (IACUC No. 13–0160).

### Subjects and harvest of oral mucosa and culture of oral mucosal epithelial cells

We collected specimens of discarded mucosal tissue after buccal mucosal transplantation surgery from subjects with (SJS, n = 3) and without (non-SJS, n = 3) SJS. All the SJS subjects were in chronic stages at least a year had passed since the occurrence. Non-SJS subjects were diagnosed with chemical burn in the eye (n = 2) or ocular malignant melanoma (n = 1). Age, sex, oral involvement and chronic ocular surface complications score (COCS; range: 0–15; scoring of conjunctival hyperemia, decreased tear volume, eyelid involvement, corneal involvement, limbal deficiency, and symblepharon formation) [14] were assessed and were compared between the groups to evaluate inter-donor demographic differences. No inter-donor demographic differences were found except in the involvement of oral mucosa of acute stage (Table 1). All three SJS subjects had oral mucosal involvement in the acute stage, while all non-SJS subjects had no oral mucosal involvement (p = 0.05, Fisher’s exact test). All the subjects did not have any oral mucosal inflammation at the time of collection of oral mucosa.

**Table 1:** Demographics of the subjects. Chronic ocular surface complications score (COCS; range: 0–15) was adapted from previous report [14].

**Demographics**	**SJS subjects**	**Non-SJS subjects**	**P value**
Sex (Male: Female)	**0:3**	**2:1**	0.4*
Age (mean±SD)	**37.3±21.8**	**53±11.5**	**0.4**^§^
COCS	**13.3±1.2**	**11.3±3.1**	**0.7**^§^
Oral mucosal involvement in acute stage (n)	**3**	**0**	0.05*
Oral mucosal inflammation at the time of collection of oral mucosa (n)	**0**	**0**	1.0*

*Fisher’s exact test

^**§**^Mann-Whitney U test

As a control, human corneoscleral rims from donor corneas provided by Northwest Lions Eye bank were obtained after penetrating keratoplasty. Human limbal epithelial cells (LEs; n = 4) were serially cultured as described in a previous report [15]. Oral mucosal epithelial cells were isolated and cultured as described by Rheinwald and Green[16]. Briefly, the culture medium was a 3:1 mixture of DMEM and F12 (GIBCO BRL, Waltham, MA, USA) containing 10% FBS and supplements as described elsewhere[17]. Mucosal epithelial cells (0.1 × 10^6^ cells/dish) were seeded on lethally irradiated 3T3 feeder cells and cultured until they reached 70~80% confluence [18, 19]. Cultured oral mucosal epithelial cell sheets (COMECs) for the *in vivo* study were grown in a 35-mm culture dish for 8~10 days and then detached from the culture dish with 1% dispase (GIBCO BRL) for 15 min at 37°C prior to transplantation.

### Colony-forming efficiency (CFE) assay

Oral mucosal epithelial cells at passage 3 or 4 were plated onto a 3T3 feeder in a 100-mm culture dish at 1 × 10^2^ cells/dish and cultured for 12 days. After the colonies were formed, the cells were fixed with 10% formaldehyde and stained with 1% rhodamine B. CFE was expressed as the percentages of the colonies formed, compared to the total number of cells inoculated. Each experiment was replicated 4 times.

### Megacolony assay

A megacolony assay was performed according to previously published procedures with certain modifications [20]. Oral mucosal epithelial cells were cultured on lethally irradiated 3T3 cells for 14 days. Colonies were scratched with a surgical blade and gently removed with a rubber policeman. The medium was aspirated and replaced with culture medium with 1 ng/ml of epidermal growth factor (EGF). The cells were then incubated for 3 days at 37°C. Images of migrating cells were captured using an inverted microscope (Olympus, Japan), and migration potential was quantified by repeatedly measuring the area from the margin of the colonies on days 1, 2, and 3. Each experiment was performed 4 times.

### Proliferation assay

Oral mucosal epithelial cells at passage 5 were plated at a density of 0.04×10^6^ cells/dish in a 100-mm culture dish and cultured for 5 days. Cells were trypsinized and counted using a hemocytometer on days 0, 2, 3, and 5. Each experiment was performed in triplicate.

### Immunofluorescence staining for oral mucosal epithelial cells *in vitro* and COMEC-transplanted corneas

Cells were fixed in a methanol-acetone solution (1:1 vol/vol), permeabilized with 0.5% Triton X-100, incubated with PBS containing 4% BSA to block non-specific binding, and stained with specific primary antibodies (1:50), including anti-human cytokeratin 3 (Progen, Heidelberg, Germany), cytokeratin 19 (Chemicon, Billerica, MA, USA), cytokeratins 1, 4, and 13, and Ki-67 (Abcam, Cambridge, MA, USA), and p63 ([4A4], Santa Cruz Biotechnology, Dallas, TX, USA), followed by the application of secondary antibodies (1:200) (FITC-conjugated donkey anti-mouse, FITC-conjugated donkey anti-rabbit, rhodamine-conjugated anti-rabbit, and rhodamine-conjugated goat anti-mouse) (Jackson Immuno Research, West Grove, PA, USA).

For analysis of COMEC-transplanted corneas, the paraffin-sectioned slides were stained with specific primary antibodies for human cytokeratin 1 (Abcam, Cambridge, MA, USA, 1:100), cytokeratin 3 (MP Biomedicals Inc., Irvine, CA, USA 1:100), cytokeratin 4 (Abcam, 1:100), cytokeratin 8 (Abcam, Cambridge, MA, USA, 1:100), cytokeratin 13 (Novocastra, UK, 1:100), and cytokeratin 19 (Chemicon, Billerica, MA, USA, 1:100), p63 (4A4; Abcam, Pre-diluted), ABCG2 (Abcam, 1:20), and Ki-67(DAKO, 1:50) were used. After incubation with rabbit anti-mouse IgG conjugated with FITC or Alexa Fluor 488 goat anti-mouse secondary antibody (Invitrogen, Eugene, USA 1:1000) at room temperature for 1 h, the slides were mounted with mounting medium. Cell nuclei were labeled with DAPI (Vector laboratories, Burlingame, CA, USA) for Ki-67 and Hoechst staining (Abcam) for p63 and visualized using a Zeiss Axioskop 40 fluorescent microscope (Zeiss, Jena, Germany). The percentage of the cells expressing p63 or Ki-67 was manually counted in 5 consecutive 400× high power fields (HPFs) by two independent blinded observers.

### ELISA

Cell lysates from limbal epithelial (n = 3) and oral mucosal epithelial (n = 6) cells were obtained by repeated cycles of freezing and thawing. The supernatant was harvested after centrifugation at 12,000 rpm for 30 min at 4°C. ELISA assay kits (R&D system, Minneapolis, MN, USA) for EGF, fibroblast growth factor (FGF), vascular endothelial growth factor (VEGF), platelet-derived growth factor (PDGF), interleukin-1α (IL-1α), transforming growth factor-α (TGF-α), TGF-β, matrix metalloproteinase-2 (MMP-2), and MMP-9 were used according to the manufacturer’s instructions. Optical density (OD) was detected at 450 nm, and data were expressed as a concentration obtained from the standard curve. All assays were performed in triplicate.

### Transplantation of the COMECs to the limbal stem cell-deficient models

A total of twelve New Zealand white rabbits, weighing 2 to 2.5 kg were anesthetized by intramuscular injection of 0.6 to 0.8 ml of a 1:1 mixture of Zoletil^™^ (1:1 tiletamine and zolazepam; virbac S.A, Nice, France) and 2% Rompun (Xylazine; Bayer, Leverkusen, Germany) and by topical proparacaine hydrochloride 0.5% (Alcaine ^**®**^, ALCON LABORATORIES, INC. Fort Worth, Texas). A cotton ball saturated with 99.9% alcohol was then applied to the rabbit corneas, including the limbus for 20 seconds. Partial thickness corneo-limbal lamellar keratectomy was performed, which included the limbus and extended 2 mm into the clear cornea. After 6–8 weeks, limbal stem cell-deficient animals showed totally conjunctivalized corneas. COMECs from three SJS subjects (5 sheets) and three non-SJS subjects (7 sheets) were transplanted into the corneas of these limbal stem cell-deficient animals. Conjunctival epithelial layers covering the rabbit cornea were removed, and a 360°peritomy was performed. The COMECs were transplanted with the epithelial side facing up and were placed on the corneal surface without any suture fixation or tissue glue. In order to enhance adherence to the corneal surface, the sheets were allowed to dry for three to five minutes. Then, one to two drops of balanced salt solution (Alcon Laboratory, Fort Worth, USA) were applied to the sheets and 18-mm soft contact lenses (Kontur PrecisionSphere, Hercules, CA, USA) were applied, and tarsorrhaphy was performed using 6–0 black silk. After surgery, 0.3% ofloxacin and 0.1% dexamethasone eyedrops were administered 4 times a day for 7 days. Intramuscular cyclosporine (25mg/day) was injected every day 1 week prior to and 1 week following surgery (for a total of 2 weeks) to reduce xenogeneic inflammation. Epithelial defects in the cornea were evaluated with 0.25% fluorescein dye using microscopy on days 3 and 7 post-surgery, and the rabbits were sacrificed on day 7. Euthanasia was carried out with intravenous pentobarbitone (85 mg/kg) in anaesthetized rabbits.

### Evaluation of the re-epithelialized area after transplantation of COMECs

Photographic images of the rabbit corneas on days 3 and 7 post-surgery were digitally analyzed using Image J software (National Institute of Health; http://imageJ.nih.gov/ij). The area of unepithelialized cornea was measured by outlining the epithelial defect using the free hand selection tool, and its relation to the total area was calculated. The percentage of the re-epithelialized corneal area was calculated by subtracting the percentage of the epithelial defect area from the total area (100%).

### Statistical analysis

GraphPad Software (GraphPad Prism, Inc., La Jolla, CA, USA) was used for statistical tests. Fisher's exact test was used for the analysis of contingency tables, and the Mann-Whitney U test was used for comparison of the means of the two groups (Demographic data for donor). The Mann-Whitney U test was used for *in vivo* comparison of p63 and Ki-67 percentage expression. With those exceptions, all other data were analyzed for a Gaussian distribution using the D’Agostino-Pearson omnibus test. To compare the means of two groups (CFE experiment), student’s t test was used, while data were analyzed by one-way ANOVA to compare the means of more than two groups (cytokine profile experiment). To analyze data affected by two factors, ordinary two-way ANOVA was used in the proliferation assay and repeated measures (RM) two-way ANOVA was used for megacolony assay and the *in vivo* re-epithelization assay. Data are presented as the mean ±SD. Differences were considered significant at P < 0.05.

## Results

### Stem cell properties of the cultured SJS and non-SJS oral mucosal epithelial cells

**Fig 1:**
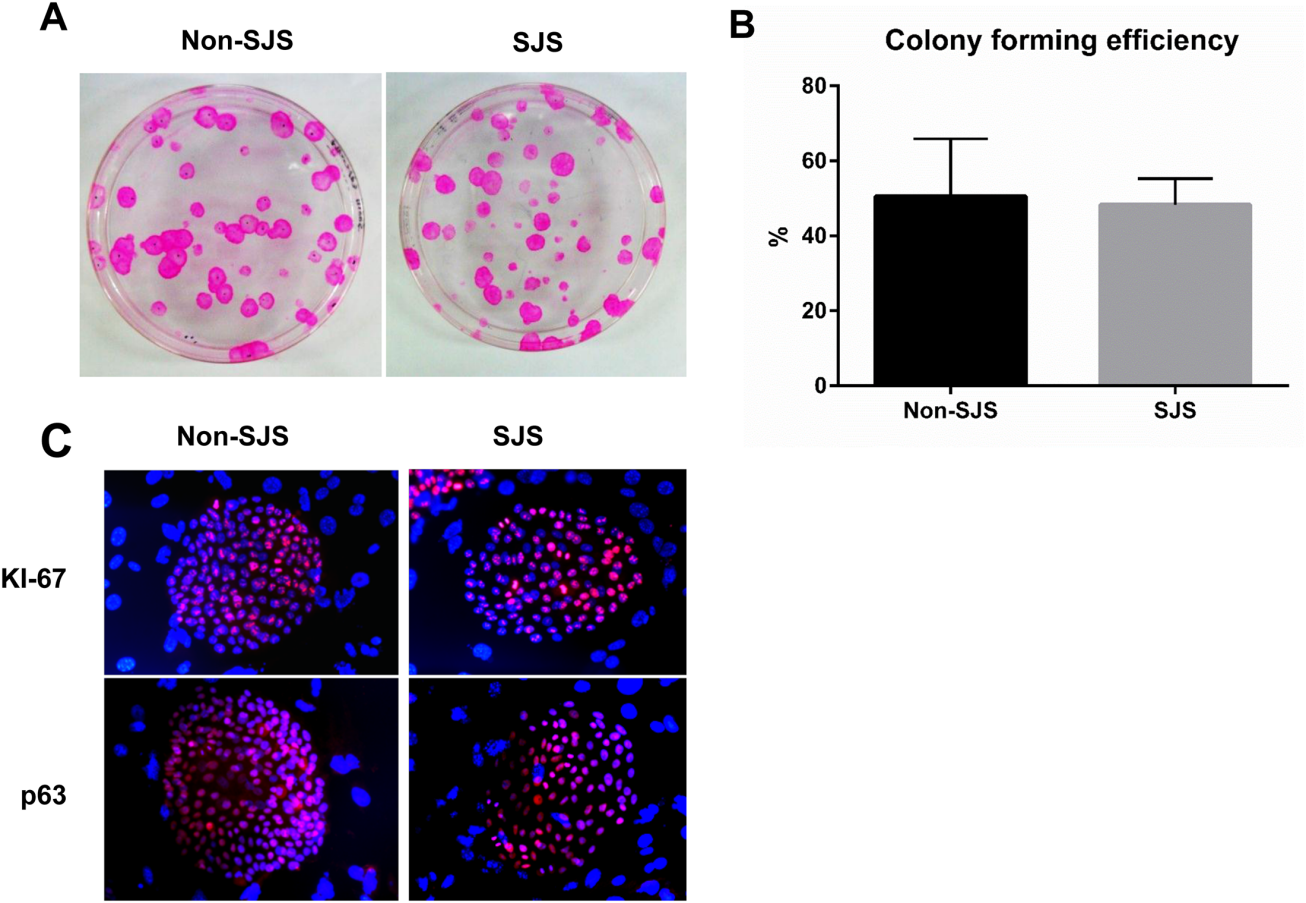
Comparison of the stem cell properties of Stevens—Johnson syndrome (SJS) and non-SJS cultured oral mucosal epithelial cells. (A) A representative independent colony-forming efficiency (CFE) experiment. (B) The CFE of SJS cells is comparable to the CFE of non-SJS cells (p>0.05, t-test). (C) High expression of proliferative and stem cell markers (Ki-67 and p63) in the non-SJS and SJS cells (X100). SJS indicates mucosal epithelial cells from SJS subjects; non-SJS indicates mucosal epithelial cells from non-SJS subjects.

In the CFE assay, most colonies of oral mucosal epithelial cells from SJS (SJS cells) or non-SJS subjects (non-SJS cells) showed holoclones, which were characterized by large colonies with smooth and circular perimeters and high growth potential. The average CFE of SJS cells (49.0%) was similar to that of non-SJS cells (51.9%) (Fig 1A and 1B; p>0.05, student’s t test). Moreover, p63, a marker for epithelial stem/progenitor cells, and Ki-67, a proliferation marker, were highly expressed in both types of cells (Fig 1C). These *in vitro* results demonstrate that both SJS and non-SJS oral mucosal epithelial cells have comparable stem cell properties.

### Proliferative and migratory capabilities of cultured SJS and non-SJS oral mucosal epithelial cells

**Fig 2:**
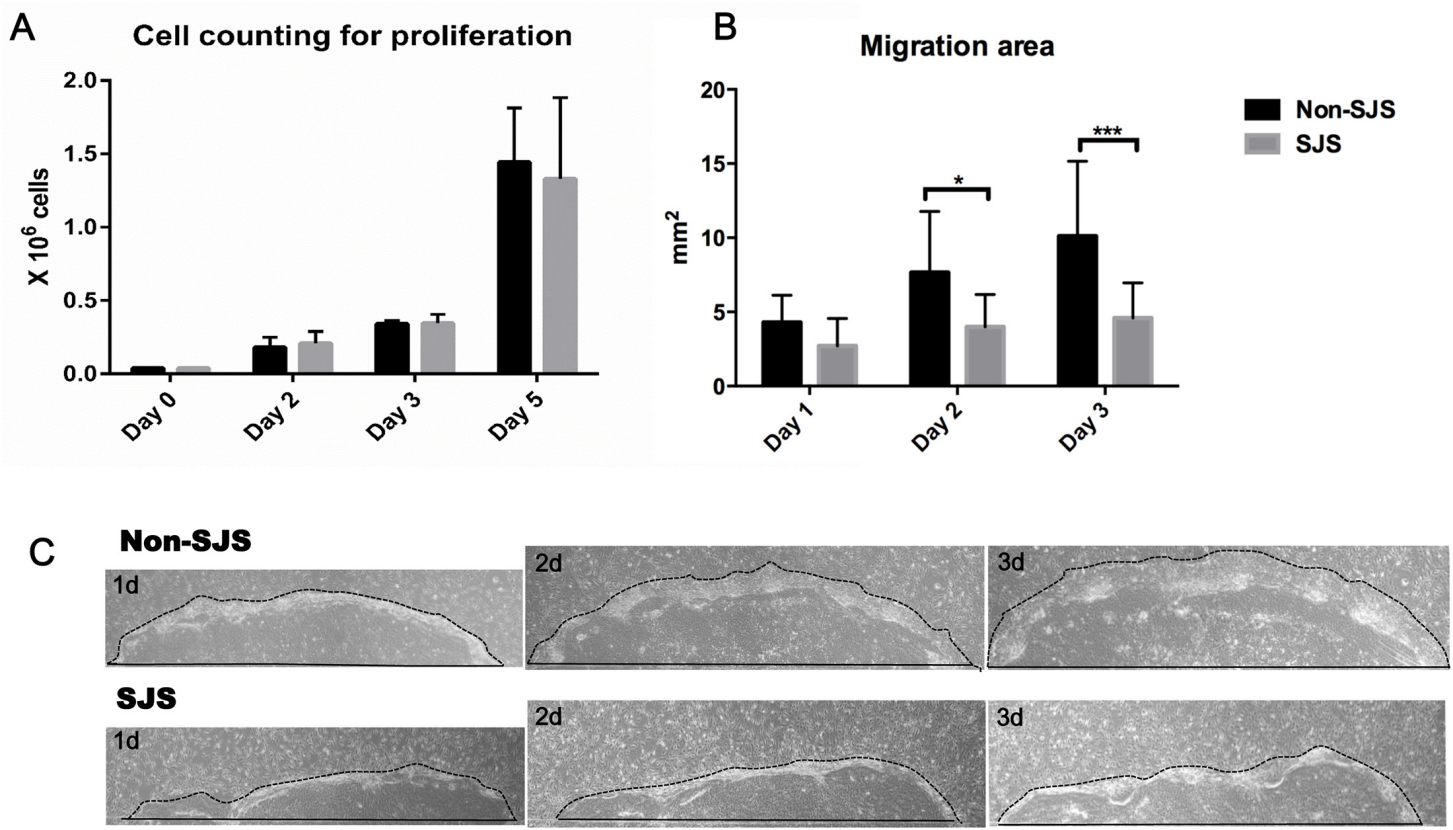
Proliferative and migratory capabilities of cultured oral mucosal epithelial cells from Stevens—Johnson syndrome (SJS) and non-SJS subjects. (A) Cells were counted on days 2, 3, and 5 and did not differ between SJS and non-SJS cells (p>0.05, two-way ANOVA). (B) Migratory potential was significantly delayed in SJS cells when compared to non-SJS cells (*p<0.05, ***p<0.001, RM two-way ANOVA). (C) Image showing cell migration from the edge (black line) (X100). The black line indicates the area that cells migrated each day. SJS indicates mucosal epithelial cells from SJS subjects; non-SJS indicates mucosal epithelial cells from non-SJS subjects.

As shown in Fig 2A, there was no difference between SJS and non-SJS oral mucosal epithelial cell proliferation after 5 days (p>0.05, two-way ANOVA), which is supported by the Ki-67 expression results shown in Fig 1C. To investigate whether there was a discrepancy in migratory potential between these cells, we performed a megacolony assay (Fig 2B and 2C). The migratory activity of SJS oral mucosal epithelial cells was significantly lower than that of the non-SJS cells on days 1 (p = 0.048, RM two-way ANOVA), 2 (p = 0.012), and 3 (p = 0.002).

### Cytokeratin expression of the SJS and non-SJS oral mucosal epithelial cells

**Fig 3:**
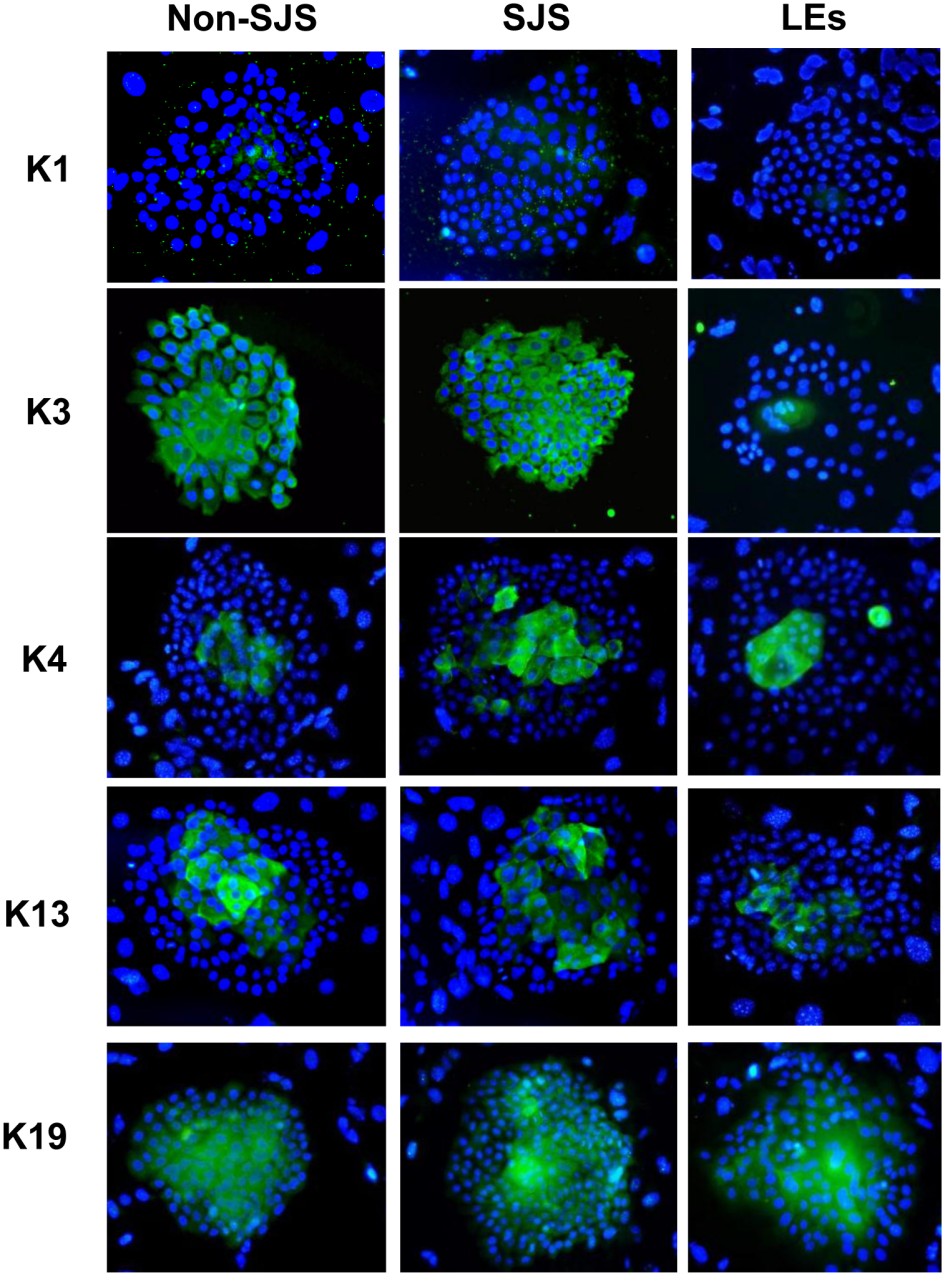
Expression of cytokeratins in oral mucosal epithelial cells and limbal epithelial (LE) cells. Cytokeratin K1 was not expressed in Stevens—Johnson syndrome (SJS) or non-SJS mucosal epithelial cells or limbal epithelial cells (LEs). K3, K4, K13 and K19 were expressed in SJS and non-SJS mucosal epithelial cells as well as LEs (X100). SJS indicates mucosal epithelial cells from SJS subjects; non-SJS indicates mucosal epithelial cells from non-SJS subjects.

K1, a marker for keratinizing stratified epithelia, was sparsely expressed in SJS and non-SJS mucosal epithelial cells, as well as in limbal epithelial (LE) cells. K3, which is present in normal center cornea, was more highly expressed in both SJS and non-SJS oral mucosal epithelial cells than in LE cells. K4 and K13, markers for non-keratinized stratified epithelia in oral mucosa, were expressed in the centers of the colonies of both cell types. K19, which is expressed in limbal stem cells or oral mucosal epithelial cells, was well expressed in both SJS and non-SJS oral mucosal epithelial cells with similar expression pattern in LE cells. These results show that both types of oral mucosal epithelial cells sustained their original phenotypes, and expressed some of the corneal phenotype during culture (Fig 3), and the phenotypes of SJS oral mucosal epithelial cells were similar to that of non-SJS oral mucosal epithelial cells.

### Cytokine profiles of the SJS and non-SJS oral mucosal epithelial cells

**Fig 4:**
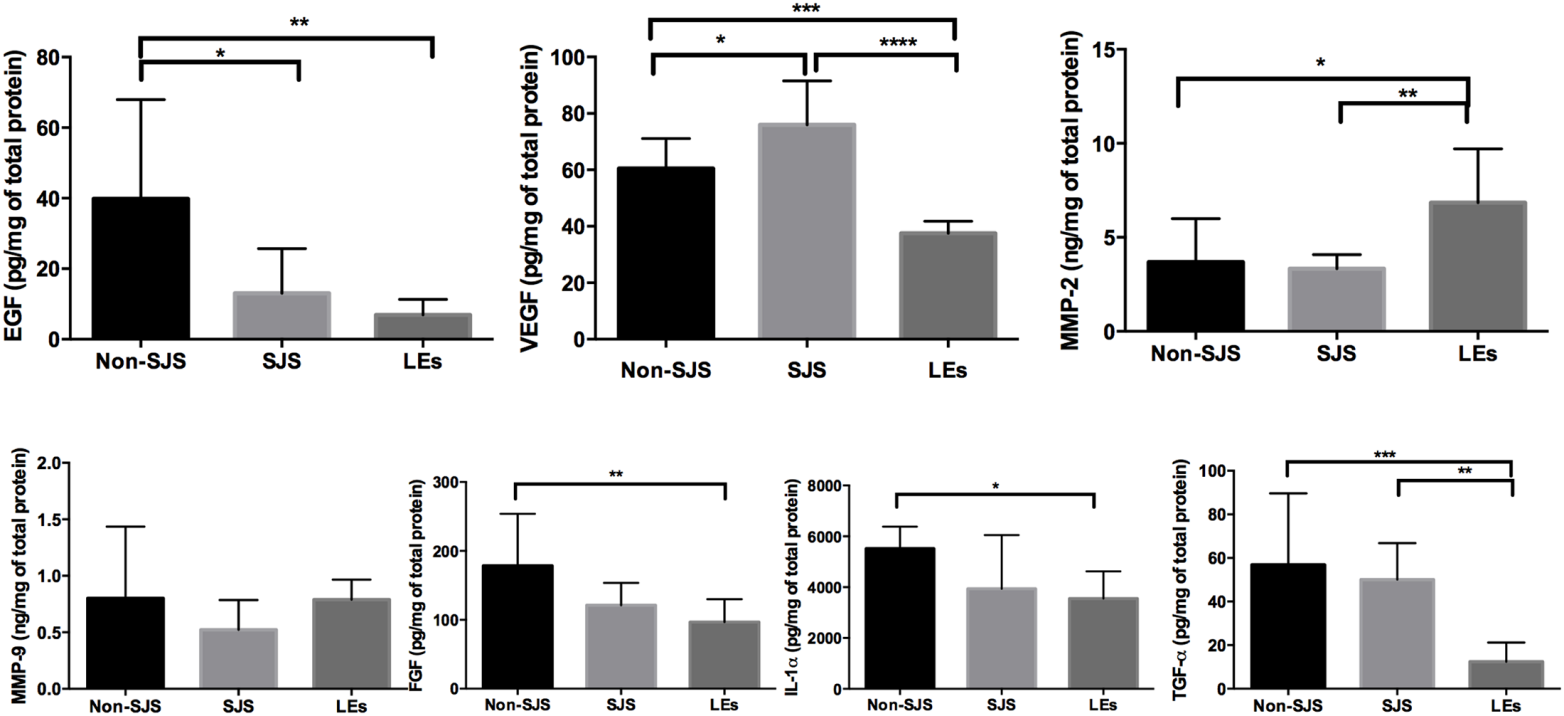
Cytokine profiles of the oral mucosal epithelial cells from Stevens—Johnson syndrome (SJS) and non-SJS subjects. ELISA assay showed that the level of VEGF was higher, and the level of EGF was lower in the cells from SJS oral mucosa (SJS) than those in the cells from normal oral mucosa (non-SJS). When compared to the expression of cytokine/growth factors in limbal epithelial cells (LEs), the level of VEGF and TFG-α were higher, while the level of MMP2 was lower in mucosal epithelial cells regardless of SJS status. The levels of FGF, EGF, and IL-1α were higher in non-SJS mucosal cells than in LEs. All data represent the mean ± standard deviation. (*p<0.05, **p<0.01, ***p<0.001, ****p<0.0001, One-way ANOVA)

Because it is thought that many different growth factors and cytokines are involved in corneal wound healing, we evaluated the levels of various cytokines in SJS and non-SJS oral mucosal epithelial cells and compared them with those in LE cells (Fig 4). The amount of VEGF in SJS mucosal epithelial cells was significantly higher than that in non-SJS mucosal epithelial cells (p = 0.019, one-way ANOVA), while the amount of EGF in SJS mucosal cells was significantly lower than that in non-SJS mucosal cells (p = 0.011). The levels of FGF, IL-1α, TGF-α, MMP-2 and MMP-9 were not significantly different in SJS and non-SJS cells.

When compared to the expression of cytokine/growth factors in LE cells, the levels of VEGF and TGF-α were higher and the level of MMP2 was lower in mucosal epithelial cells (p<0.05) regardless of the donor SJS status. FGF, EGF, and IL-1α were more highly expressed in non-SJS mucosal cells (p<0.05) than in LE cells. PDGF and TGF-β were undetectable in mucosal epithelial cells, regardless of SJS status.

### *In vivo* surface reconstruction of SJS- and non-SJS-COMECs

**Fig 5:**
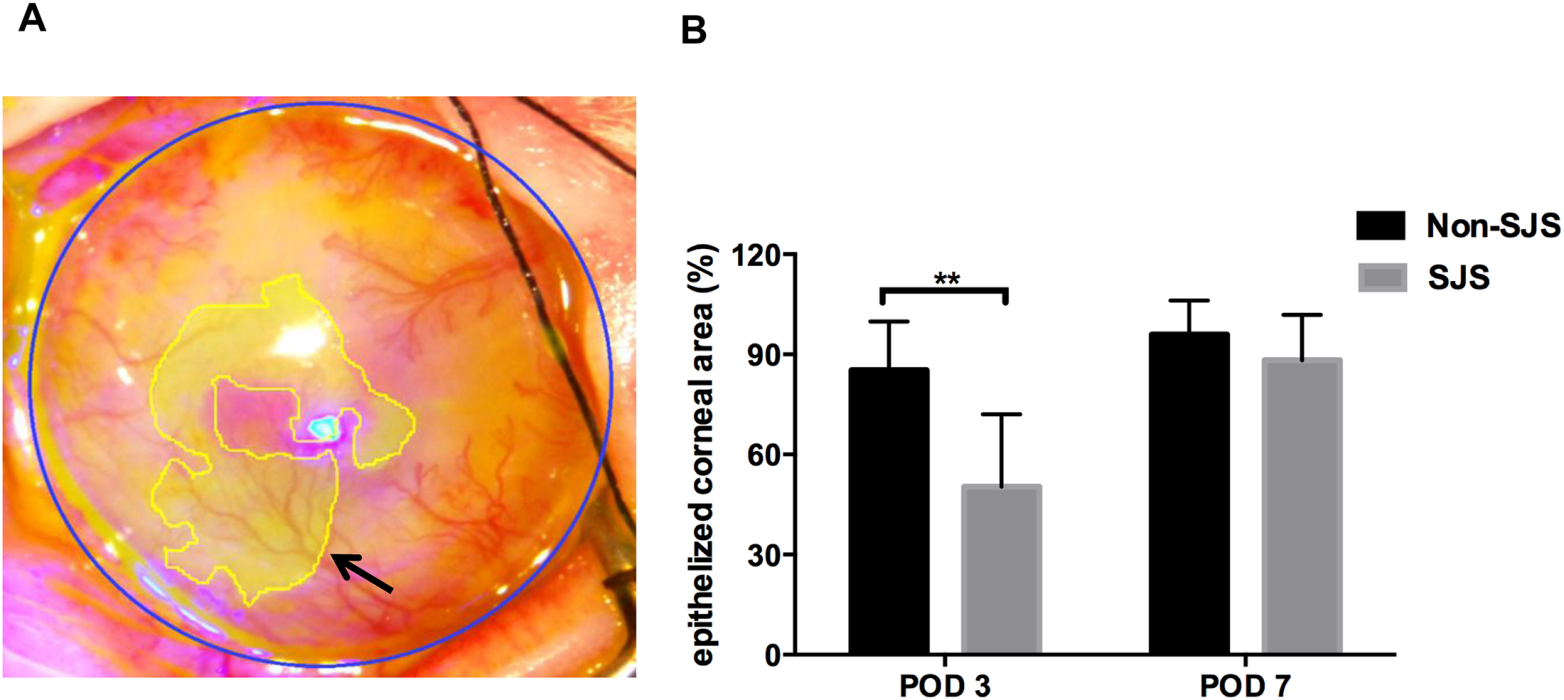
*In vivo* re-epithelialization by Stevens—Johnson syndrome (SJS) and non-SJS cultured oral mucosal epithelial cell sheets (COMECs) in a limbal stem cell-deficient model. (A) Digital measurement of the epithelial defect area (black arrow) and calculation of re-epithelialized corneal area. (B) The re-epithelialized area was smaller in SJS-COMECs than in non-SJS-COMECs at postoperative day (POD) 3 (**p<0.01, RM two-way ANOVA) but was not statistically different between SJS and non-SJS-COMECs on POD 7. SJS indicates cells from SJS subjects; non-SJS indicates cells from non-SJS subjects.

We grossly examined the re-epithelialization potential of SJS and non-SJS COMECs after transplantation into limbal stem cell-deficient rabbits (Fig 5). The area of corneal epithelial defects was larger in the eyes transplanted with COMECs from SJS subjects than in the eyes with COMECs from non-SJS subjects on day 3 (p = 0.007, RM two-way ANOVA). It suggests that the migratory potential of SJS COMECs may be initially delayed. However, there was no difference in the area of corneal epithelial defects on day 7.

### *In vivo* characteristics of cytokeratin and stemness in SJS- and non-SJS-COMECs

**Fig 6:**
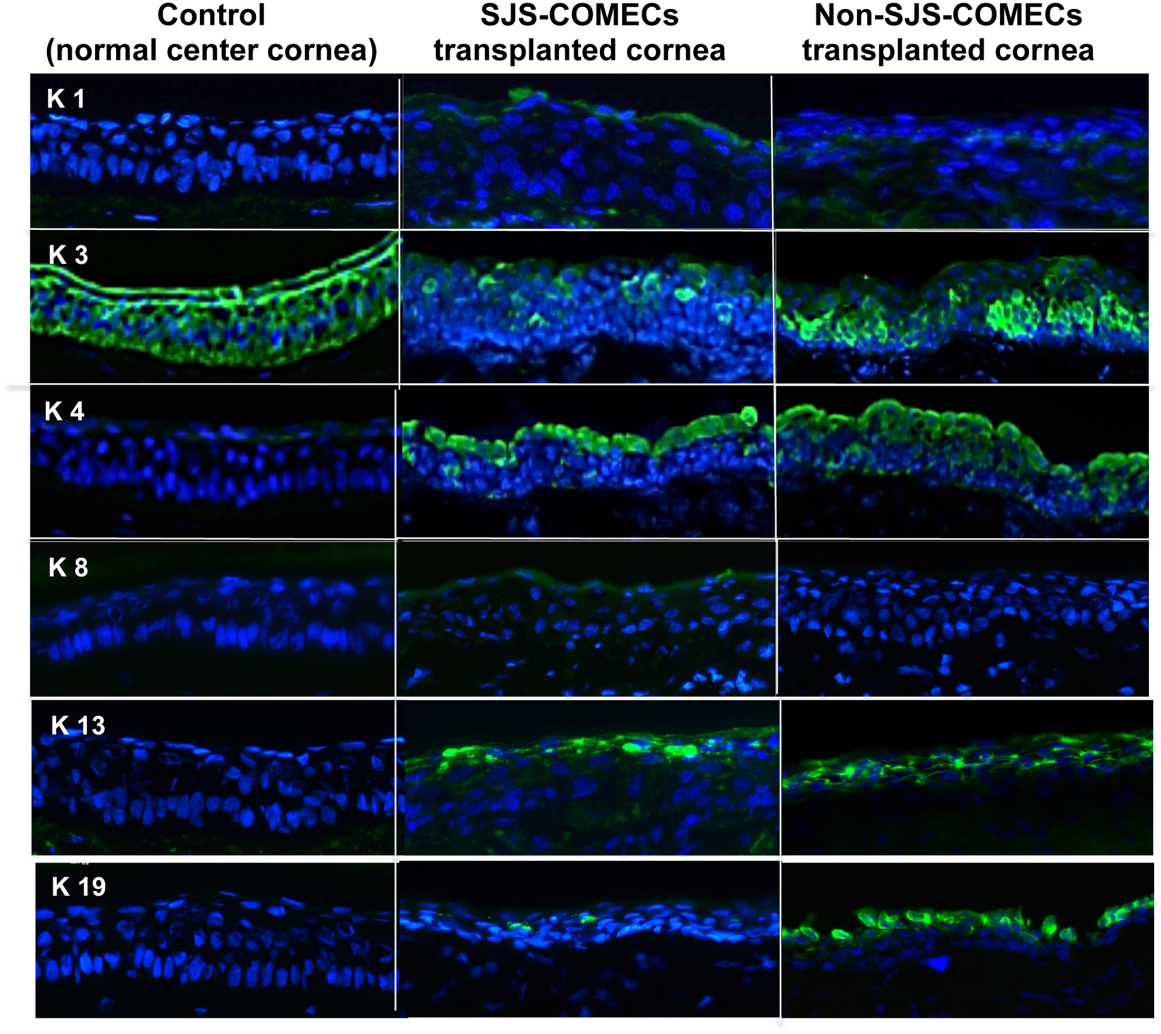
Epithelial characteristics of the ocular surface transplanted with Stevens—Johnson syndrome (SJS) and non-SJS COMECs on day 7 post-transplantation. Corneas with COMECs from both SJS and non-SJS subjects expressed K3, K4 and K13 (X200). K1 and K8 were not expressed in both groups. K19 was expressed in cornea transplanted with non-SJS COMECs, and it was barely expressed in cornea transplanted with SJS COMECs. The control indicates a normal center cornea.

On day 7 post-transplantation, the corneas with SJS- and non-SJS-COMECs had similar expression levels of K3, as a marker for differentiated corneal epithelium, K4 and K13, as mucosa-specific keratins (Fig 6). It suggests that both COMECs from SJS and non—SJS subjects maintain corneal phenotype as well as phenotypes of oral mucosal cells. On the other hand, K19 as a marker for oral mucosal epithelium was barely expressed in SJS-COMECs transplanted cornea compared with that in non-SJS-COMECs transplanted cornea, despite similar expression of K19 in *in vitro* colonies of both groups prior transplantation. K1, as a marker for keratinized epithelium and K8, as a marker for secretory epithelium such as conjunctival epithelial cells, both of which are not expressed in normal oral mucosa were not expressed in both COMECs-transplanted corneas.

**Fig 7:**
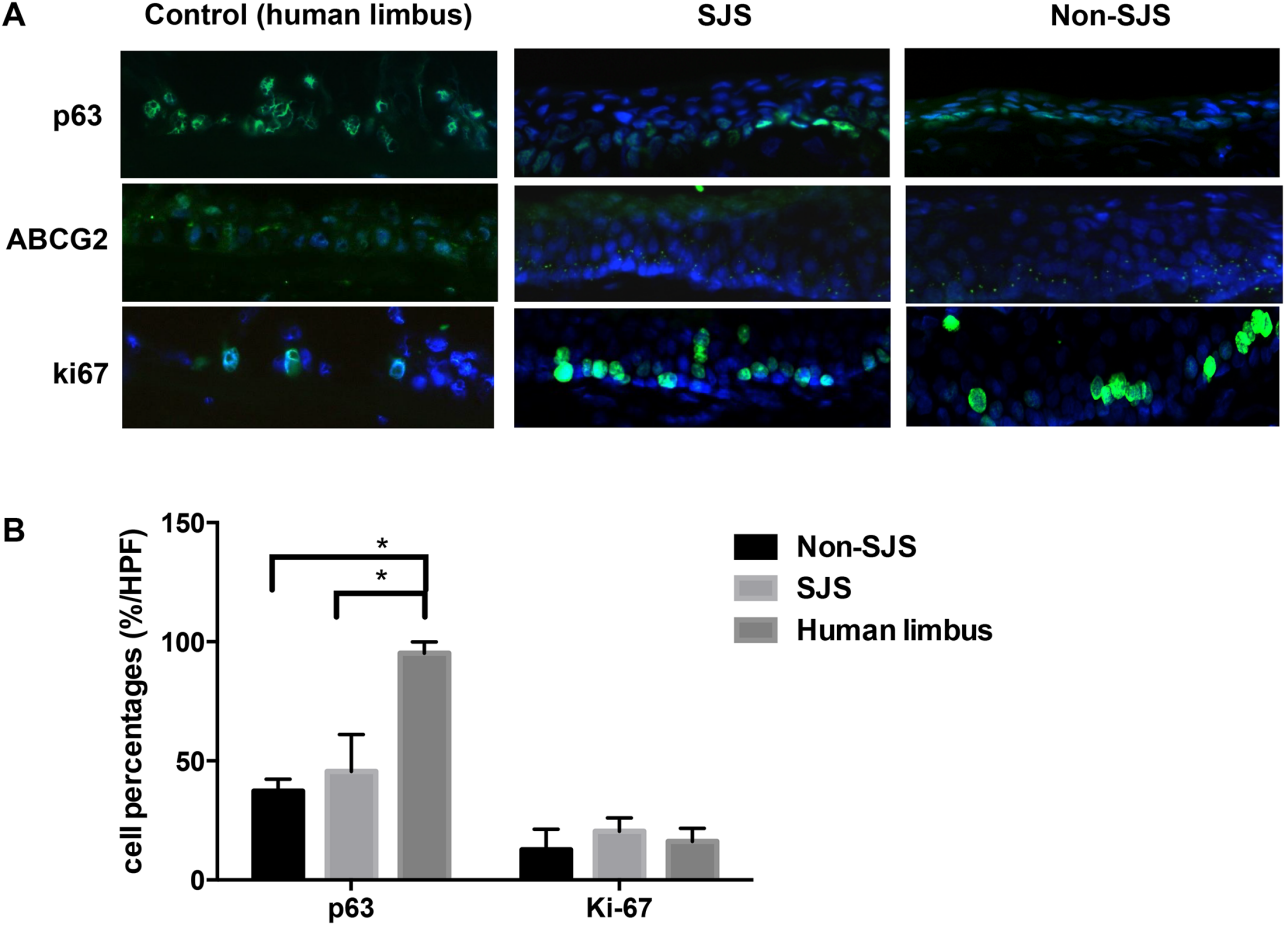
Characteristics of the progenitor cell expression with Stevens—Johnson syndrome (SJS) and non-SJS COMECs on day 7 post-transplantation. (A) Cornea with COMECs from both SJS and non-SJS subjects highly expressed p63, ABCG2, and Ki-67 (X400). (B) Percentage of p63- or Ki-67-positive cells in the corneas transplanted with SJS-COMECs was not different from that in the corneas transplanted with non-SJS-COMECs (p>0.05, Mann-Whitney U test). The percentage of p63 was lower in both COMECs-transplanted corneas from SJS and from non-SJS subjects than that in normal human limbal tissues as control (p<0.05, Mann-Whitney U test). The control indicates normal limbal tissues of human.

P63, ABCG2, and Ki-67 (Fig 7A) were well expressed in both groups, suggesting that COMECs from both groups have similar stem cell potentials. The percentage of p63- or Ki-67-positive cells in corneas transplanted with SJS-COMECs was not significantly different from that in corneas transplanted with non-SJS-COMECs (Fig 7B; p>0.05, Mann-Whitney U test). The percentage of p63 was lower in both COMECs-transplanted corneas from SJS and from non-SJS subjects than that in normal human limbal tissues as control (Fig 7B; p<0.05, Mann-Whitney U test).

## Discussion

Our results demonstrated that the stem cell properties, proliferation potential, keratin expression, and for the most part, cytokine expression of cultured human oral mucosal epithelial cells from SJS subjects were comparable to those from non-SJS subjects. Although the mucosal epithelial cells from SJS subjects had a lower migratory potential, the initial delay in wound healing observed in corneas treated with SJS-COMECs had disappeared by day 7 because of comparable stem cell function.

Although transplantation of autologous cultured limbal or oral mucosal epithelial cell sheets offers substantial treatment for patients with intractable limbal stem cell deficiency[6, 13, 21, 22]. clinical reports of success in achieving stable ocular surfaces vary (57.5~100%)[23]. Long-term success depends on host factors as well as transplanted cell factors[24]. Host factors include the severity of ocular damage, presence of lid abnormalities, presence of symblepharon, severity of the tear film impairment, and the level of inflammation, which depends on the severity of the primary disease. Transplanted cell factors include the proportion of stem cells in the sheets, cell sources (i.e., limbal epithelial cells, oral mucosal epithelial cells, or nasal epithelial cells), and primary characteristics of the cells that depend on the underlying systemic disease. A recent report showed that disease-specific outcomes with persistent epithelial defects frequently occurred in the eyes of SJS patients and pathologic keratinization was common in the eyes of patients with ocular pemphigus[12]. There is a concern that clinical outcomes may be affected not only by factors of the receiving cornea but also by the functions of the donor cells being modified under chronic inflammation in cases of pemphigus or by acute inflammation in SJS. For that reason, we examined cell characteristics in light of the underlying primary diseases. Fortunately, the general characteristics and the proportion of SJS mucosal epithelial stem cells from the donors who had an oral inflammation in acute stage seem to be preserved *in vitro* and *in vivo* in a manner comparable with mucosal epithelial stem cells from non-SJS subjects without any inflammation in oral mucosa. *In vitro* and *in vivo* differentiation, as measured by cytokeratin 3 expression, was similar in SJS and non-SJS cells. The expression of cytokeratin 4 and 13 from both groups were comparable in *in vitro* colonies and *in vivo* corneas. However, *in vivo* expression of cytokeratin19 in SJS-COMECs transplanted corneas was lower than that in non-SJS-COMECs transplanted corneas, although colonial expression of cytokeratin19 was similar in both groups. This suggests that some of the mucosal characteristics may be partially changed in epithelial cells from SJS subjects when the cells are placed under the harsh environment like limbal stem cell-deficient eyes. So far, we do not know why there is a difference in the pattern of CK19 expression between *in vitro* culture and *in vivo* transplanted sheet. Differential expression of cytokeratin 19 between those two should be further investigated in future. Negative staining of cytokeratin 1 and cytokeratin 8 indicated that COMECs-transplanted cornea was not contaminated by skin or conjunctival epithelial cells.

Suprisingly, we found that initial migration of SJS mucosal epithelial cells was delayed *in vitro* and *in vivo* after transplantation with SJS-COMECs. This might be, in part, caused by a low capability of the mucosal epithelial cells in SJS to secrete EGF as well as MMP-2, both of which influence the migration of epithelial cells[25, 26]. Considering that all three SJS donors showed oral involvement, the EGF-secreting capacity of oral mucosal epithelial cells may be altered by the disease processs. Our data suggests that commercially available EGF recombinant may have be beneficial in ameliorating delayed wound healing after transplantation with SJS-COMECs. A comparable proliferative potential in mucosal epithelial stem cells of SJS overcame delayed wound healing by day 7, despite a slightly different cytokine profile from that of non-SJS cells. This suggests that COMECs from SJS subjects may function as well as COMECs from non-SJS subjects.

Another interesting finding was that SJS oral mucosal epithelial cells expressed high levels of VEGF compared to either non-SJS mucosal cells or limbal epithelial cells. Following cultivated autologous oral mucosal epithelial transplantation (COMET), various degrees of corneal neovascularization have been observed, which indicate more neovascularization compared with that in ocular surfaces reconstructed with autologous limbal epithelial transplantation[6, 27–29]. A recent report supported this idea by demonstrating a lack of anti-angiogenic factors in ocular surface reconstructed with COMET[30]. Given that neovascularization is a concern for ocular surfaces reconstructed with COMET, increased expression of VEGF in SJS cells suggests that ocular surface reconstructed with COMECs from SJS subjects may show more neovascularization when compared to surfaces reconstructed with COMECs from healthy subjects.

There are several limitations to this study. The availability of human tissues was limited, and therefore, the sample size was small. The *in vivo* studies were performed using a human-to-rabbit xenogeneic transplantation model. Consequently, we could not evaluate the long-term stabilization, reduction of neovascularization and characterization of the COMECs although systemic immune suppression was administered. However, to our knowledge, this is the first study to characterize and compare oral mucosal epithelial cells from SJS subjects with those of non-SJS subjects, and we believe that understanding the regenerative potential of COMECs from SJS subjects is a first vital step in treating SJS-related limbal stem cell deficiency.

## Supporting Information

S1 Appendix Transfer technique of COMECs on the cornea. (AVI)
